# Knee valgus and patellofemoral instability after pediatric anterior cruciate ligament reconstruction: a case report and review of the literature

**DOI:** 10.1186/s13256-023-03920-2

**Published:** 2023-05-22

**Authors:** Jamison G. Gamble, Rati N. Shirodkar, James G. Gamble

**Affiliations:** 1 St. George’s University School of Medicine, St. George’s, Grenada West Indies; 2 Department of Orthopaedic Surgery, Stanford University School of Medicine, Stanford Children’s Health, Stanford, CA 94304-5341 USA

**Keywords:** Pediatric ACL reconstruction, Valgus knee deformity, Patellofemoral instability, Case report

## Abstract

**Background:**

Pediatric athletes who undergo anterior cruciate ligament reconstruction are at risk for a growth deformity if the surgery violates the physes.

**Case:**

A 12-year-old African American boy underwent anterior cruciate ligament reconstruction using a hamstring autograft. The procedure violated the distal femoral growth plate and the perichondrial ring of LaCroix, resulting in a distal femoral lateral physeal growth arrest. Three years later, he had developed a 15° valgus deformity, an increased quadriceps angle and patellofemoral instability. He was able to return to sports after undergoing a distal femoral osteotomy to correct the valgus and medial patellofemoral ligament reconstruction to stabilize the patella.

**Conclusion:**

Anterior cruciate ligament reconstruction in athletes with open physes has the potential to cause distal femoral valgus deformity, an increased quadriceps angle, and subsequent patellofemoral instability.

## Introduction

Anterior cruciate ligament (ACL) reconstruction in pediatric patients has increased over the last decade as the frequency of these injuries has increased in the pediatric population [1–8]. Many factors have been associated with the increased frequency of ACL injuries, including the increased intensity of sports participation, the increased volume (time) of sports participation, and the increased early specialization of pediatric athletes [5]. Both physeal sparing and transphyseal procedures have been advocated for ACL reconstruction in skeletally immature athletes. However, procedures that violate open growth plates have the potential to cause growth deformities.

The purpose of this communication is to report the case of a pediatric athlete who underwent ACL reconstruction and developed a distal femoral valgus deformity that contributed to recurrent patella dislocations. The patient and his parents were informed that the data concerning this case would be submitted for publication, and they provided consent.

## Case report

A 15-year-old African American boy sustained his third left lateral patella dislocation while running and rapidly changing directions (cutting) during a football game. His other two dislocations happened in the same non-contact manner during the previous year. Three years prior to presentation, the patient had undergone ACL reconstruction at another institution using a hamstring autograft with metaphyseal suspension on the femoral side.

On physical examination, he had focal pain to palpation along the medial aspect of the left patella and a large effusion. He demonstrated apprehension when the patella was gently pushed laterally. The Lachman examination had a firm endpoint with ~5 mm excursion. The quadriceps angle (Q-angle) measured with a goniometer, was 15° on the left and 5° on the uninvolved right. His left lower extremity was 1.5 cm shorter than his right.

**Fig. 1:**
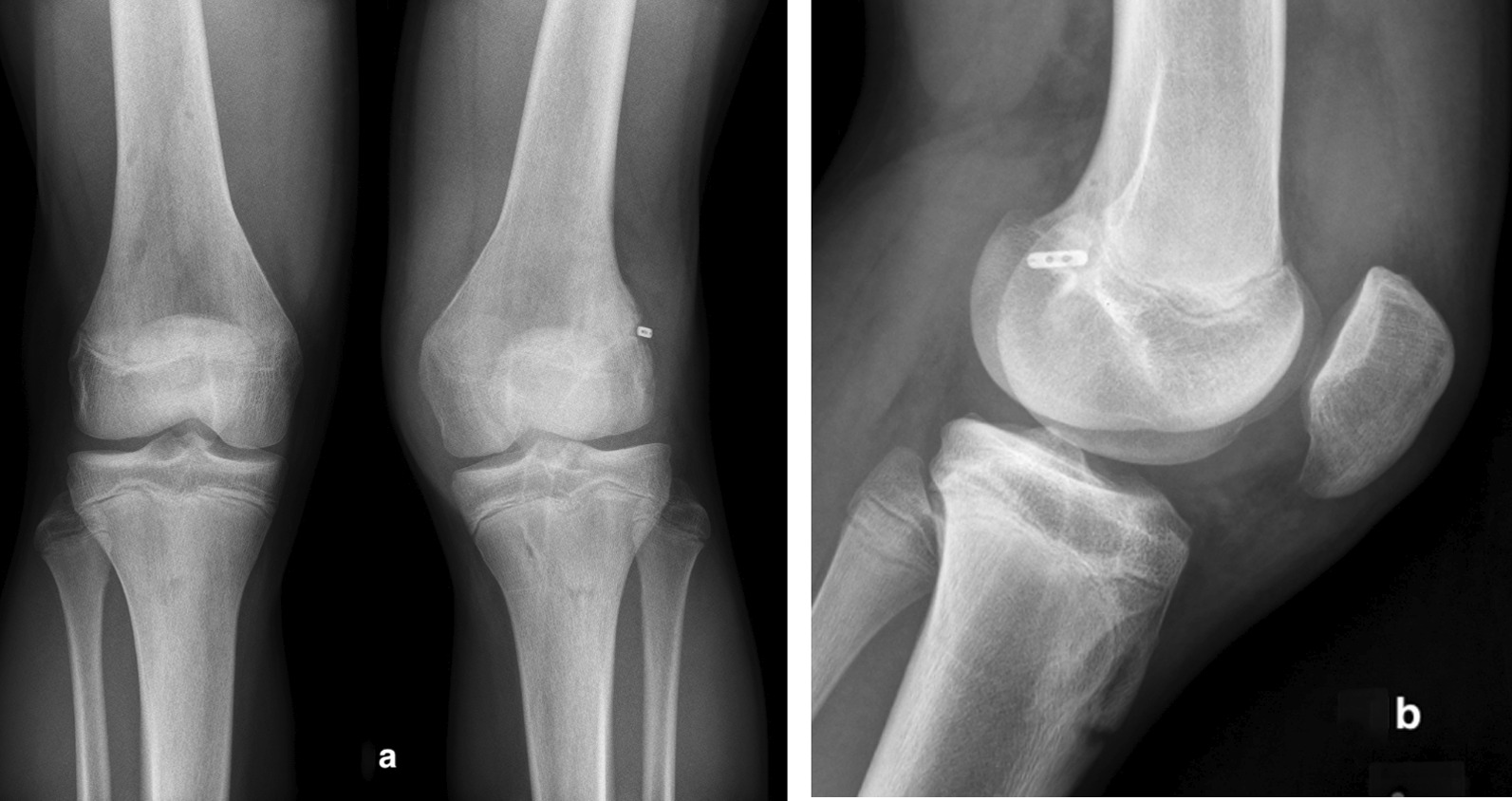
**a** and** b** Anteroposterior (**a**) and lateral (**b**) radiographs showing the metallic suspension device at the left distal femoral lateral cortex, 15° of valgus of the left knee with tilting of the joint space

The radiograph showed premature closure of the lateral aspect of the distal femoral physis and the presence of a metallic suspension button along the lateral edge of the left distal femoral cortex (Fig. 1a and b). The anatomic axis of the knee, as measured on our institution’s picture archiving and communication system (PACS), was 15° on the left and 5° on the right.

**Fig. 2:**
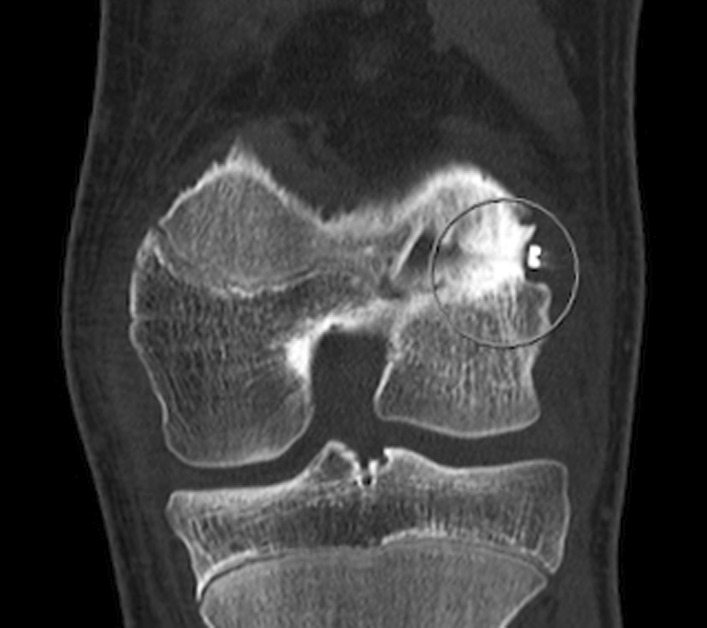
Coronal CT scan showing sclerosis with closure of the lateral aspect of the distal femoral physis and the location of the metallic button over the peripheral aspect of the growth plate (circle) where it had subsided into the bone

A computed tomographic (CT) scan (Fig. 2) confirmed closure of the lateral aspect of the distal femoral physis with the metallic suspension button located directly over the area of the perichondrial ring of LaCroix and subsided below the cortex.

**Fig. 3:**
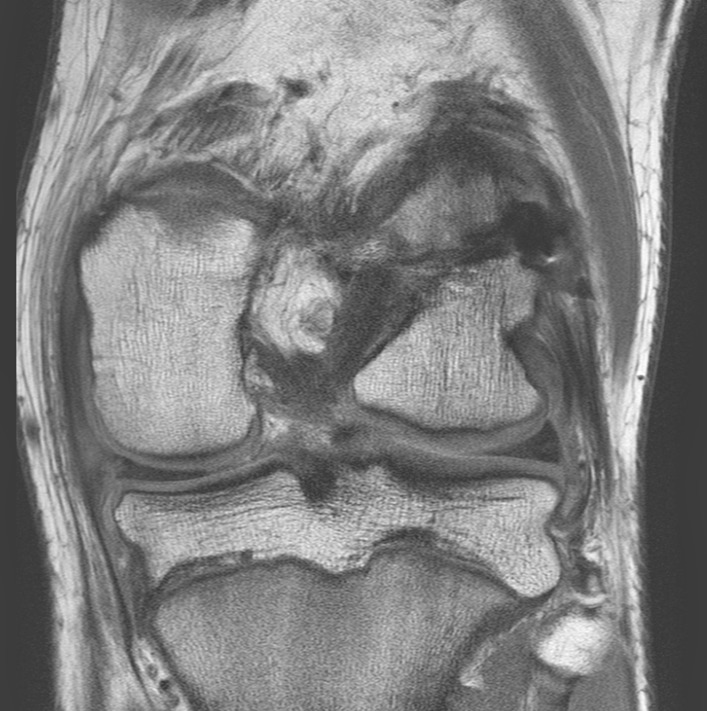
Coronal T1 weighted MRI scan showing decreased signal intensity within the lateral aspect of the distal femoral where the 10 mm tunnel had been made

Magnetic resonance imaging (MRI) showed the presence of a 10 mm area of decreased signal intensity throughout the posterior-lateral aspect of the physis (Fig. 3). The medial patellofemoral ligament (MPFL) had been completely avulsed from the medial edge of the patella.

**Fig. 4:**
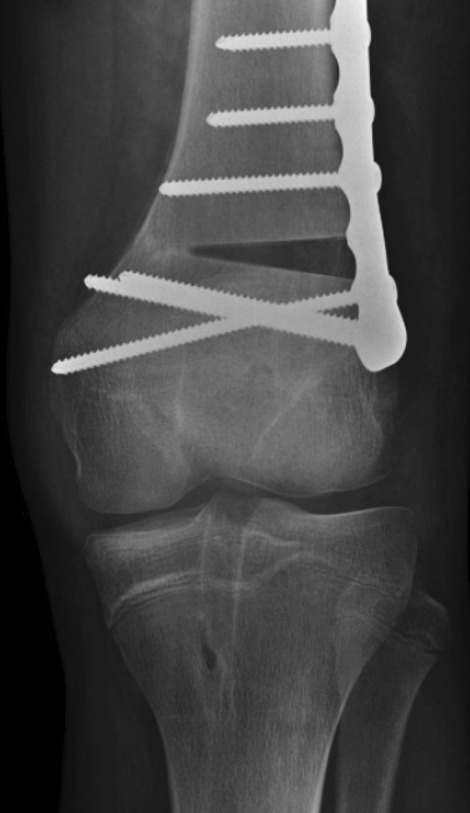
Left distal femoral opening wedge osteotomy that was used to correct the valgus

He underwent a distal femoral opening wedge osteotomy to correct the valgus (Fig. 4).

**Fig. 5:**
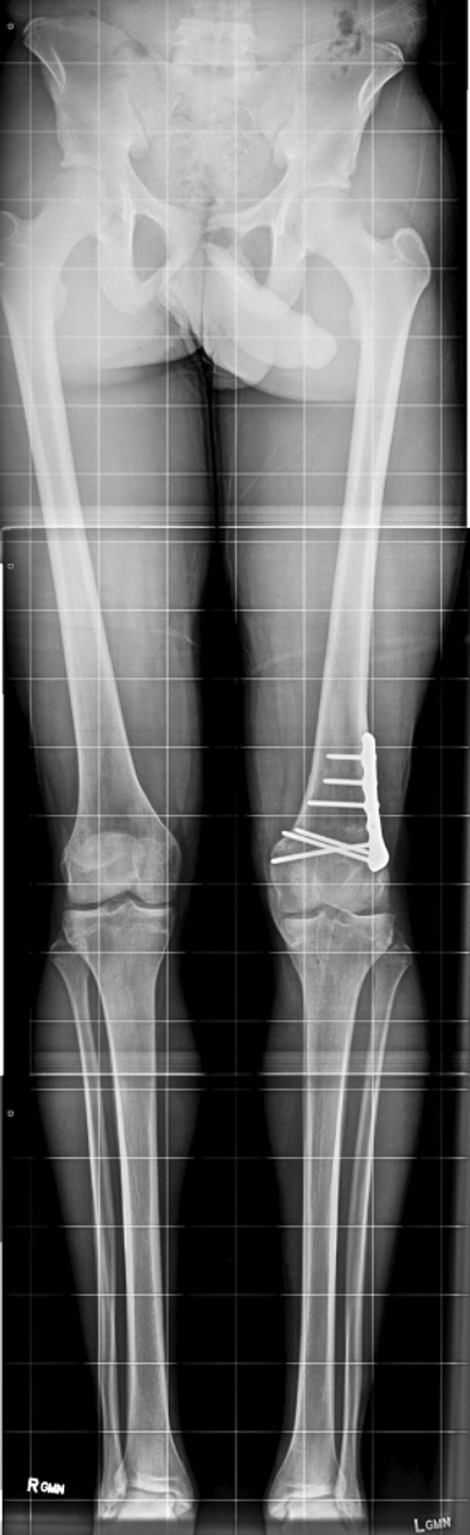
Latest radiograph after returning to full sports activities showing improvement of alignment, but with a 1.2 cm shortening of the left lower extremity as measured on our institutions PACS

Six months later, he underwent a medial patellofemoral ligament reconstruction using a tibialis posterior tendon allograft. At the latest evaluation, 4 years after the MPFL reconstruction (Fig. 5), he was asymptomatic and had returned to full sports activity. The last radiograph (Fig. 5) showed improved alignment with a 1.5 cm limb length discrepancy on the left side and slight joint space narrowing.

## Discussion

ACL tears have increased in the pediatric population during the last decade [3, 5]. Studies have shown that active ACL deficient children and adolescents are at increased risk for meniscus injury, chondral injury, and early degenerative articular changes [9]. As a result, surgeons recommend ACL reconstruction in active children with open growth plates. Both physeal sparing and transphyseal procedures have been advocated. A survey by Kocher *et al.* [10] found that 88% of responding surgeons had treated an ACL deficient skeletally immature patient during the past year, of which 78% had performed ACL reconstructions with a mean patient age of 11.5 years. The survey also found that 79% preferred a transphyseal technique on the tibial side, and 68% preferred a transphyseal technique on the femoral side. Eleven percent of respondents reported that they had seen growth disturbances from ACL reconstruction, with 80% of the disturbances involving the femoral side and 20% the tibial side. Across all respondents, they found eight cases of distal femoral valgus with arrest and two cases without arrest.

Published reports of growth disturbances after pediatric ACL reconstruction are increasing [11–15]. Wong, Feeley, and Pandya [12] performed a metaanalysis review of complications after pediatric ACL reconstruction. They found a total of 58 growth disturbances reported in the English literature, of which 16 required surgeries. Thirty-seven patients developed limb length discrepancies. Twenty-one knees developed angular deformity, 14 valgus, 2 flexion, 2 varus, and 3 recurvatum, including 3 combined coronal and sagittal deformities. The same authors [13] published another review that compared outcomes of two physeal sparing techniques (over-the-top and all-epiphyseal). The review summarized data from 10 studies, with a total of 482 knees from 478 skeletally immature patients. The authors noted that both physeal-sparing techniques were successful in addressing the ACL deficiency, and both techniques had a similar frequency of growth disturbances. Limb overgrowth was the most common postoperative complication overall and was more commonly found in patients who had reconstruction with the all-epiphyseal technique, while angular deformities were found in the group that received reconstruction via the over-the-top technique. Rates of rerupture were similar in both groups. In the over-the-top group, there were three total angular deformities, one of which was a 4° valgus and another a 4° varus; neither required surgical correction. The third patient with angular deformity had undergone the over-the-top reconstruction. He developed a multiplanar flexion-valgus deformity with 13° of valgus, 9° of flexion, and 1 cm of shortening, which required a multiplanar distal femoral osteotomy. Anderson and Anderson [14] also reviewed studies on ACL reconstruction in skeletally immature athletes. They confirmed that reports of ACL injuries in the pediatric population have been increasing, and ACL reconstructions have increased as well. These authors referred to papers concerning three cases of growth abnormalities following physeal-sparing procedures, one of which was a distal femoral valgus deformity from bone-bridge formation after using the over-the-top surgical technique. They speculated that complications can be due to direct damage to the growth plate during surgery, but also due to indirect damage from the graft causing a soft tissue tethering of the growth plate.

Collins *et al.* [15] performed a systematic literature review of deformities and limb length discrepancies following ACL reconstruction in skeletally immature patients. They found 21 studies reporting 39 patients with growth abnormalities; 62% of the growth abnormalities were limb overgrowth. These authors noted that physeal-sparing techniques had been used in 47% of limb length discrepancy cases and 25% of angular deformity cases. They also mentioned that the incidence of growth abnormalities post ACL reconstruction in skeletally immature patients is probably grossly underreported.

Liddle *et al.* [17] performed transphyseal ACL reconstructions using four-stranded hamstring tendon grafts in 17 patients who were Tanner stage 1 or 2 and noted only one instance of 5° valgus, which resulted in no functional impairment. Likewise, McCarthy and Harty [18] performed transphyseal ACL reconstructions with hamstring graft utilizing tibial and femoral tunnels with diameters ranging from 7 to 9 mm. They reported no incidence of leg length discrepancy greater than 1 cm, but they did find all patients averaged together had a valgus of 0.8° ± 2.4° with a range of 5° valgus to 3° varus. Holwein *et al.* [19] examined transphyseal ACL reconstructions with hamstring grafts using one of three metaphyseal femoral fixation techniques: Endobutton (*n* = 19), Rigidfix pins (*n* = 14) or bioabsorbable interface screws (*n* = 4). The authors noted that in the four cases utilizing screws, the screws had been positioned proximal to the physis. After a mean follow-up of 24.9 months, the authors noted that compared with the non-operated knees, the operated knees utilizing screw fixation had a higher degree of valgus (1.9° ± 1.5°) compared with Endobutton (0.5° ± 1.7°, *p* = 0.37) or pin (0.8° ± 1.6°, *p* = 0.44). Shifflett *et al.* [20] performed transphyseal ACL reconstruction with hamstring graft on, four patients (two boys and two girls), and they noted two cases of recurvatum and two cases of valgus with angles of 3.8° and 8.0°. Lemaitre et al. [16] emphasized that the tunnel diameter must not exceed 8 mm and should be as vertical and central as possible to prevent valgus. Table 1 summarizes the reported cases of distal femoral valgus deformities after pediatric ACL reconstruction.

**Table 1:** Reported cases in the English literature involving instances of distal femoral valgus following ACL reconstruction

Study type	Reconstruction technique	Graft type	Patient age	Cases of valgus	Degrees of valgus	References
Case report	Transphyseal with Leeds-Keio artificial ligament	Artificial	6 years	1	20°	21
Case series	MacIntosh and Nakhostine with iliotibial band	Auto	10 years 5 months	1	5°	25
Case series	Transphyseal with hamstring	Auto	12 years 1 months	1	5°	17
Case series	Physeal sparing with hamstring	Auto	12 years 6 months^†^	11	1.05°^†^ (0.1°–2.3°)^‡^	24
Case series	Transphyseal with hamstring	Auto	13 years 2 months^†^ (boys) 13 years 1 months^†^ (girls)	37	1.9° ± 1.5°^†§^ (screw fixation, *n* = 19) 0.5° ± 1.7°^†§^ (button, *n* = 14) 0.8° ± 1.6°^†§^ (pin, *n* = 4)	19
Case report	Transphyseal with hamstring	Auto	13 years 7 months	2	4°	16
Case series	Transphyseal with hamstring	Auto	14 years 2 months	2	3.8° (*n* = 1) 8.0° (*n* = 1)	20
Case series	Transphyseal with hamstring	Auto	14 years 4 months^†^	22	0.8° ± 2.4°^†§^ (maximum = 5°)	18
Case report	Transphyseal with hamstring	Allo	14 years 4 months	1	14.0°	22

^†^Average

^‡^Range

^§^Standard deviation

Our 15-year-old African American boy patient developed a distal femoral valgus deformity with lateral physeal arrest and a secondary patellofemoral instability after undergoing ACL reconstruction when he was 12-years-old. Advanced imaging 3 years after the operation confirmed the presence of a 10 mm tunnel going through the lateral aspect of the physis and the presence of a metallic suspension button located over the perichondrial ring of LaCroix that had subsided into the bone. These two situations most likely caused the physeal damage and the subsequent distal femoral valgus of 15°, resulting in patellofemoral instability. We thought it best to do an opening wedge osteotomy, given his limb length discrepancy prior to the MPFL reconstruction.

## Conclusions

It is important to document complications after surgical treatment of pediatric patients undergoing ACL reconstruction. The complication in this case was a 15° valgus deformity and subsequent patellofemoral instability. A 10 mm tunnel had been drilled horizontally through the lateral aspect of the physis, and the metaphyseal suspension button had damaged the perichondrial ring of LaCroix. Valgus knee alignment is one of the known causes of patellofemoral instability, recurrent patella dislocations, and damage to the MPFL, requiring surgical reconstruction [27–29]. Most postoperative valgus deformities reported in the literature were clinically insignificant. In the case reported here, the 15° iatrogenic valgus deformity was clinically significant, contributing to the patellofemoral instability.

At the latest evaluation, 4 years after correction of the valgus and reconstruction of the MPFL, the patient was asymptomatic and had returned to full sports participation. However, the long-term prognosis is unclear as the patient does have a 1.2 cm limb length discrepancy, a slight varus alignment, and evidence of early joint space narrowing that may predispose the patient to osteoarthritis [30].

## Data Availability

Not applicable.

## Abbreviations

ACL: Anterior cruciate ligament
Auto: Autograft
Allo: Allograft
CT: Computed tomographic scan
MPFL: Medial patellofemoral ligament
MRI: Magnetic resonance imaging
PACS: Picture archiving and communication system
Q-angle: Quadriceps angle
